# Identification of osteosarcoma m6A-related prognostic biomarkers using artificial intelligence: RBM15

**DOI:** 10.1038/s41598-023-28739-1

**Published:** 2023-03-31

**Authors:** Jie Jiang, Haishun Qu, Xinli Zhan, Dachang Liu, Tuo Liang, Liyi Chen, Shengsheng Huang, Xuhua Sun, Jiarui Chen, Tianyou Chen, Hao Li, Yuanlin Yao, Chong Liu

**Affiliations:** 1 The First Clinical Affiliated Hospital of Guangxi Medical University, Nanning, 530021 People’s Republic of China; 2 Department of Traditional Chinese Medicine, The People’s Hospital of Guangxi Zhuang Autonmous Region, Nanning, 530016 People’s Republic of China

**Keywords:** Bone cancer, Cancer genetics, Cancer genomics, Cancer models, Oncogenes, Tumour biomarkers, Tumour immunology

## Abstract

Osteosarcoma has the worst prognosis among malignant bone tumors, and effective biomarkers are lacking. Our study aims to explore m6A-related and immune-related biomarkers. Gene expression profiles of osteosarcoma and healthy controls were downloaded from multiple public databases, and their m6A-based gene expression was utilized for tumor typing using bioinformatics. Subsequently, a prognostic model for osteosarcoma was constructed using the least absolute shrinkage and selection operator and multivariate Cox regression analysis, and its immune cell composition was calculated using the CIBERSORTx algorithm. We also performed drug sensitivity analysis for these two genes. Finally, analysis was validated using immunohistochemistry. We also examined the RBM15 gene by qRT-PCR in an in vitro experiment. We collected routine blood data from 1738 patients diagnosed with osteosarcoma and 24,344 non-osteosarcoma patients and used two independent sample *t* tests to verify the accuracy of the CIBERSORTx analysis for immune cell differences. The analysis based on m6A gene expression tumor typing was most reliable using the two typing methods. The prognostic model based on the two genes constituting RNA-binding motif protein 15 (RBM15) and YTDC1 had a much lower survival rate for patients in the high-risk group than those in the low-risk group (P < 0.05). CIBERSORTx immune cell component analysis demonstrated that RBM15 showed a negative and positive correlation with T cells gamma delta and activated natural killer cells, respectively. Drug sensitivity analysis showed that these two genes showed varying degrees of correlation with multiple drugs. The results of immunohistochemistry revealed that the expression of these two genes was significantly higher in osteosarcoma than in paraneoplastic tissues. The results of qRT-PCR experiments showed that the expression of RBM15 was significantly higher in both osteosarcomas than in the control cell lines. Absolute lymphocyte value, lymphocyte percentage, hematocrit and erythrocyte count were lower in osteosarcoma than in the control group (P < 0.001). RBM15 and YTHDC1 can serve as potential prognostic biomarkers associated with m6A in osteosarcoma.

## Introduction

Osteosarcoma, a highly malignant skeletal tumor that develops primarily in children and adolescents, can be treated using neoadjuvant chemotherapy and surgery; however, it still has a high potential for local recurrence and metastasis^1,2^. It has also been shown that m6A promotes the self-renewal of tumor stem cells and the proliferation and differentiation of tumor cells^3^. It is owing to the poor prognosis of osteosarcoma and the associated physical and emotional trauma that exploring osteosarcoma biomarkers related to prognosis should be prioritized.

N6-methyladenosine (m6A) epitranscriptional modification is considered to be the most conserved and abundant internal transcriptional modification, particularly in eukaryotic RNA (mRNA). m6A is mainly recognized by m6A methylesterases (METTL3/14, WTAP, RNA-binding motif protein 15 [RBM15]/15B, VIRMA, and ZC3H13); demethylases (FTO, ALKBH5, and ALKBH3); and m6A binding proteins (YTHDC1/2, YTHDF1/3, IGF2BP1/3, HNRNP, and eIF3)^4^. In recent years, more and more studies on m6A in cancer have reported that β-catenin stimulates m6A modification and subsequent translation of HSF1 mRNA by inhibiting miR455-3p production; thus, targeting HSF1 may be a potential therapeutic strategy to intervene in cancers^5^. Rui Su et al. demonstrated that genetic depletion of FTO Alpha-Ketoglutarate Dependent Dioxygenase (FTO) and drug inhibition largely attenuated the self-renewal of leukemic stem cells and inhibited the gene expression of immune checkpoint genes, and reprogrammed the immune response; thus, making cancer therapy targeting FTO a potential therapeutic modality^6^. Thus, m6A methylation modifications occupy a highly important position in tumors; however, their specific role in osteosarcoma is largely unknown.

The tumor microenvironment (TME) comprises mainly of immune cells and tumor cells that infiltrate the tumor mixed with stromal components^7^. Notably, the immune profile within the tumor is an important factor that can affect patient survival and response to immunotherapy^8^. Moreover, the ability of tumor cells to continuously interact with their microenvironment and the tumor microenvironment composition has been demonstrated to block immune checkpoint responses, in addition to the intrinsic biomarkers of tumor cells. Moreover, a growing body of research suggests that TME plays an integral role in both guiding the patient’s medications and treatment^9^. The tumor microenvironment limits the immune response, and T cells must initiate an appropriate immune response by consuming large amounts of nutrients. In contrast, TME may be metabolically unfavorable owing to insufficient vascular exchange and cancer cell metabolism, leading to nutrient depletion and accumulation of waste products^10^. In this study, we used CIBERSROT software to quantify the immune cell typing of the osteosarcoma gene expression matrix to explore the relationship between immune cells and osteosarcoma; thus, guiding the clinical use of drugs.

In recent years, research on genes and disease prognosis has received increasing attention from scholars and is gaining attention from researchers as a new method to predict patient prognosis^11–13^. Since the specific mechanisms of m6A methylation modification in osteosarcoma are not fully understood, the main objective of this study was to explore prognostic biomarkers associated with m6A methylation modification as well as immune correlation through a combination of bioinformatics and experiments in order to guide clinical diagnosis, treatment and predict prognosis.

## Materials and methods

### Data download and preliminary processing

The gene expression data for osteosarcoma used for analysis were downloaded through the University of California Santa Cauz (UCSC Xena, http://xena.ucsc.edu/) database, which was synchronized using the cancer genome atlas (TCGA, https://www.cancer.gov/about-nci/organization/ccg/research/structural-genomics/tcga) database. This is updated simultaneously, making it more convenient for researchers to download and analyze the data. In contrast, the gene expression data of normal tissues used for analysis were downloaded from the Genotype-Tissue Expression Project (GTEx, https://www.genome.gov/Funded-Programs-Projects/Genotype-Tissue-Expression-Project). GSE21257 was downloaded from the Gene Expression Omnibus (GEO, https://www.ncbi.nlm.nih.gov/geo/query/acc.cgi?acc=GSE21257^14^) database as a validation cohort. In this study, all statistical analyses, as well as bioinformatics image plotting, were performed using the programming language R (version R x64 4.0.2, https://www.r-project.org/). The downloaded data were normalized and processed using log2.

### Extraction of m6A-related genes, differential expression analysis, and correlation analysis

In this study, we extracted m6A-related genes for a deeper study to analyze the role of m6A-related genes in osteosarcoma. We extracted 13 m6A-related genes using the “limma” package^15^. Subsequently, we performed differential expression analysis of these genes with cut off values set to |logFC| > 0.15, P < 0.05. We also visualized these genes as heat maps using the “pheatmap” package. We used the “reshape2” and “ggpubr” package^16^ to correlate and visualize these genes to understand their relationships.

### Gene oncology enrichment analysis (GO) and protein–protein interaction network

To further explore the role of m6A-related genes in osteosarcoma, we used the “colorspace,” “stringi,” “ggplot2,” “digest,” “GOplot,” “clusterProfiler,” and “org.Hs.eg.gg” packages. The “org.Hs.eg.db” and “enrichplot” packages are used for GO enrichment analysis. Also, we imported these m6A-related genes into the String database (https://www.string-db.org/) to obtain the protein–protein interaction network, which was then visualized using Cytoscape 3.8.0.

### m6A-related tumor typing and its correlation analysis

In this study, tumor typing was first performed based on the gene expression of m6A. We used the “ConsensusClusterPlus” and the “limma” packages to perform tumor typing analysis. Nine different fractal methods were simulated to determine the best value of the cumulative distribution function (CDF), which is the integral of the probability density function. Subsequently, after determining the optimal CDF values, tumor typing was performed, and the principal component analysis was visualized using the “limma” and “ggplot2” packages. CDF, also known as distribution function, is an integral of probability density function, and its main function is to completely describe the probability distribution of a real random variable X^17^. Here, we use it to analyze the most suitable for fractalizing tumor into several clusters. Subsequently, the survival analysis was plotted based on different clusters to analyze the relationship between m6A-related tumor typing and prognosis. We combined and analyzed the clinical information with that of tumor staging to obtain clinical information related to tumor staging. Finally, we also performed a survival analysis of the patients in the two cluster groups.

### Construction of m6A-related prognostic model for osteosarcoma

To analyze the relationship between the m6A-related genes and prognosis, the Least absolute shrinkage and selection operator (LASSO) and multivariate COX regression analysis method, respectively was used to construct the prognostic models. The LASSO method can achieve the effect of variable selection by compressing the coefficients of insignificant variables to zero. Although the ridge method also compresses the original coefficients to some extent, none of the coefficients are compressed to zero, and the final model retains all the variables. Therefore, LASSO regression is a complex yet accurate method to streamline the model. The LASSO regression method was used to obtain the minimum penalty coefficient for constructing the model. Subsequently, a multivariate COX regression analysis was used to analyze survival status and survival time together with multiple factors of gene expression to derive genes for the construction of the prognostic model, with a cut-off value set at P < 0.05. To test our model, we have validated our model using the dataset GSE21257 downloaded from the GEO database as a validation dataset. The model was constructed using the same method for GSE21257. We used the “survival” package and the “survminer” package to analyze the forest maps that visualized the genes used to build the model. We obtained a risk score for each patient, as well as the mean value of the patient’s risk score, and divided all patients with osteosarcoma into a high-risk or low-risk group.

### Examination of m6A-related prognostic models for osteosarcoma

To test the accuracy of our constructed m6A-related osteosarcoma prognostic model, the “survivor”, “survminer”, and “timeROC” packages were used to construct ROC diagnostic curves for 1, 3, and 5 years for the prediction model. In contrast, we used the “ggpubr” package to analyze the differences in gene expression of the constructed models in the high and low-risk groups.

### Survival analysis

In this study, the survival data were fully analyzed using different analytical methods to make the most of the valuable osteosarcoma case data. First, based on their expression, the “survival” package was used to separate each m6A-related gene into high and low m6A-related gene expression groups, and the differences in their KM curves were analyzed. Second, a prognostic model was constructed for osteosarcoma, and based on this with high and low-risk groups, the survival differences between these groups were analyzed.

### Prognostic prediction and clinical correlation analysis of m6A-related osteosarcoma prognostic model

After constructing a prognostic model for osteosarcoma, the “rms” package was used to construct 1-year, 3-year, and 5-year calibration curves and nomogram plots to predict the survival rate of patients with osteosarcoma. The calibration curve, a plot of actual versus predicted incidence, is essentially a visualization of the results of the Hosmer–Lemeshow goodness-of-fit test and is an effective method to evaluate the consistency of the Cox regression model. The nomogram graph is a scoring system that adds up all the scores and finally corresponds to the probability of survival. The subgroups were constructed based on high and low-risk groups, and the relationship of clinical information was analyzed using these two genes.

### Immune cell composition analysis

To investigate the relationship between m6A-related genes and immune cells in osteosarcoma, a quantitative analysis was performed of their immune cell content using CIBERSROT software^18^. The latter extracted the expression of genes in immune cells as a reference label, used a linear model to predict the immune cell content in the tumor, and assessed significance in the results using a permutation test. Here, the immune cell content of all osteosarcoma samples was assessed and analyzed using P < 0.05 as a screening index.

### Drug sensitivity analysis

Here, to analyze the relationship between these two genes and drug sensitivity, we downloaded the gene expression files and drug sensitivity files from the CellMiner database (Version: 2021.1, database:2.6) database. The CellMiner database is based on 60 cancer cells listed through the National Cancer Institute's Center for Cancer Research (NCI), and the NCI-60 cell line is the most widely used cancer cell sample population for anticancer drug testing today^19,20^. The gene expression files and drug sensitivity files were downloaded from the CellMiner database (Version: 2021.1, database:2.6), and were analyzed using the "impute" package, the "limma" package, the "ggplot2" package, and the "ggpubr The gene expression and drug sensitivity analyses were carried out using the "impute", "limma", "ggplot2" and "ggpubr" packages.

### Immunohistochemistry

To further validate the reliability of our analysis at the experimental level, a study was performed using immunohistochemistry methods. All pathological tissue sections used for immunohistochemistry were obtained from patients diagnosed with osteosarcoma requiring surgical treatment at the First Clinical Affiliated Hospital of Guangxi Medical University. This study was in accordance with the Declaration of Helsinki of the World Medical Assembly, and the study was approved by the Ethics Department of the First Clinical Affiliated Hospital of Guangxi Medical University, approval number: 2021 (KY-E-087). We performed immunohistochemical analysis on specimens from anonymous patients, which did not require informed consent from the patients according to the approval of the ethics department of the First Clinical Affiliated Hospital of Guangxi Medical University. RBM15-specific antibody was purchased from Proteintech (https://www.ptgcn.com/, Catalog number: 10587–1-AP) and diluted 1:200 for staining; YTHDC1-specific antibody was purchased from Abcam (https://www.abcam.cn/products/primary-antibodies/ythdc1-antibody-epr21821-213-ab259990.html) and diluted 1:500 for staining. The http://www.Abcam.cn/, catalog number: ab259990, was diluted 1:500 for staining. All pathological tissue sections were subjected to dewaxing, hydration, antigen repair, blocking of endogenous peroxidase, serum closure, primary antibody incubation, detection system incubation (secondary antibody), DAB development, re-staining, alcohol dehydration, and sealing before placing under an inverted microscope for observation. Then, the positive rate of stained areas was assessed for all stained pathological sections using Image J (V 1.8.0) software (https://imagej.en.softonic.com/), and statistical analysis was performed using a *t* test for two-paired sample means with IBM SPSS Statistics 25. Finally, the results of the positive rate were visualized using Prism 8 (V 8.0.0, https://www.graphpad.com/guides/prism/8/user-guide/) software.

### Quantitative reverse transcription PCR (q-RT-PCR)

Fluorescence real-time quantitative PCR is used to quantify the starting template by monitoring the fluorescence signal of the product of each cycle in the amplification reaction in real time and analyzing the amplification of the exponential phase. Here, the normal human osteoblasts hFOB1.19, human osteosarcoma cells mnng and human osteosarcoma cells HOS used were purchased from Shenzhen Aowei Biotechnology Co (http://www.otwobiotech.com/). We used the NCBI homepage (http://www.ncbi.nlm.nih.gov/) for gene sequence lookup and Primer premier 5.0 for primer design. We performed cell culture of all cells purchased, and after obtaining enough cells, we performed RNA extraction from two types of osteosarcoma cells and control cells. Subsequently, real-time quantitative PCR was performe. The details of primer design are shown in Table 1.

**Table 1:** Primer sequences of GAPDH and RBM15.

Primer	Sequence (5′–3′)
RBM15-F	CTGCCTGAGGAGAGTGGAGGAC
RBM15-R	CGGCTACTGCTCAATTCTGGACTG
GAPDH-F	CCACTCCTCCACCTTTGAC
GAPDH-R	ACCCTGTTGCTGTAGCCA

### Big data blood test immune cell discrepancy

Here, we collected routine blood data from screened non-osteosarcoma patients and osteosarcoma patients attending the First Affiliated Hospital of Guangxi Medical University between January 1, 2010 and January 1, 2022 for testing immune cell differences in order to test the accuracy of immune cells obtained from CIBERSORTx software (https://cibersortx.stanford.edu/) calculations. This study was in accordance with the Declaration of Helsinki of the World Medical Assembly, and the study was approved by the Ethics Department of the First Clinical Affiliated Hospital of Guangxi Medical University, approval number: 2021 (KY-E-087).We performed statistical analysis of these two groups using two independent samples *t* test. The statistical results were also visualized using the programming language R.

### Ethical disclosure

This study was approved by the Ethics Review Committee of the First Clinical Affiliated Hospital of Guangxi Medical University and was in accordance with the Declaration of Helsinki of the World Medical Associatio.

## Results

### Data download and preliminary processing

**Figure 1:**
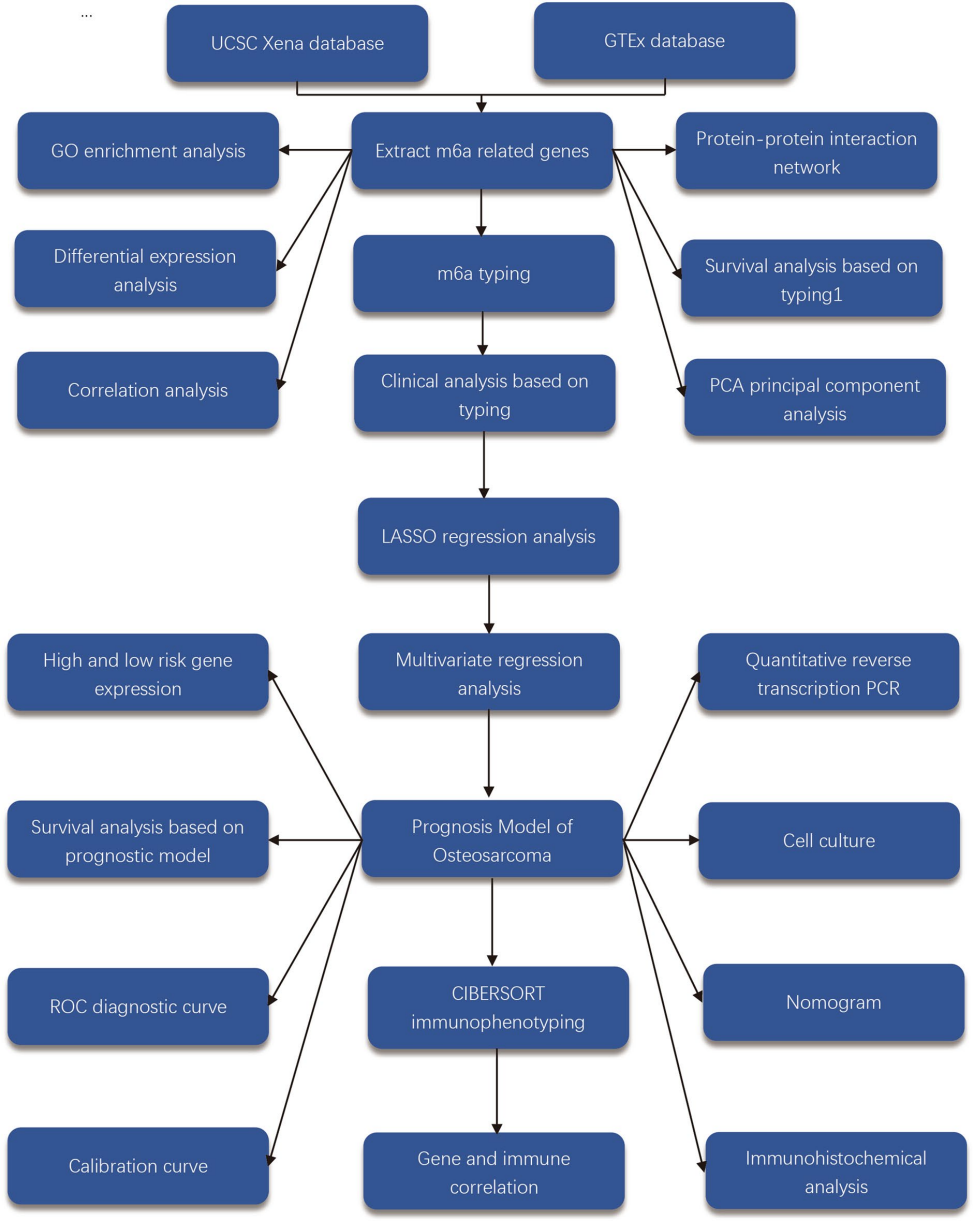
Flowchart. This figure shows the workflow diagram of this study.

The gene expression data were downloaded from the UCSC Xena database for 88 patients with osteosarcoma, while 396 normal samples were downloaded from the GTEx database as normal controls. We normalized the downloaded data, and log2 processed all the data. The steps of the work were plotted as a flow chart (Fig. 1).

### Extraction of m6A-related genes, differential expression analysis, and correlation analysis

**Figure 2:**
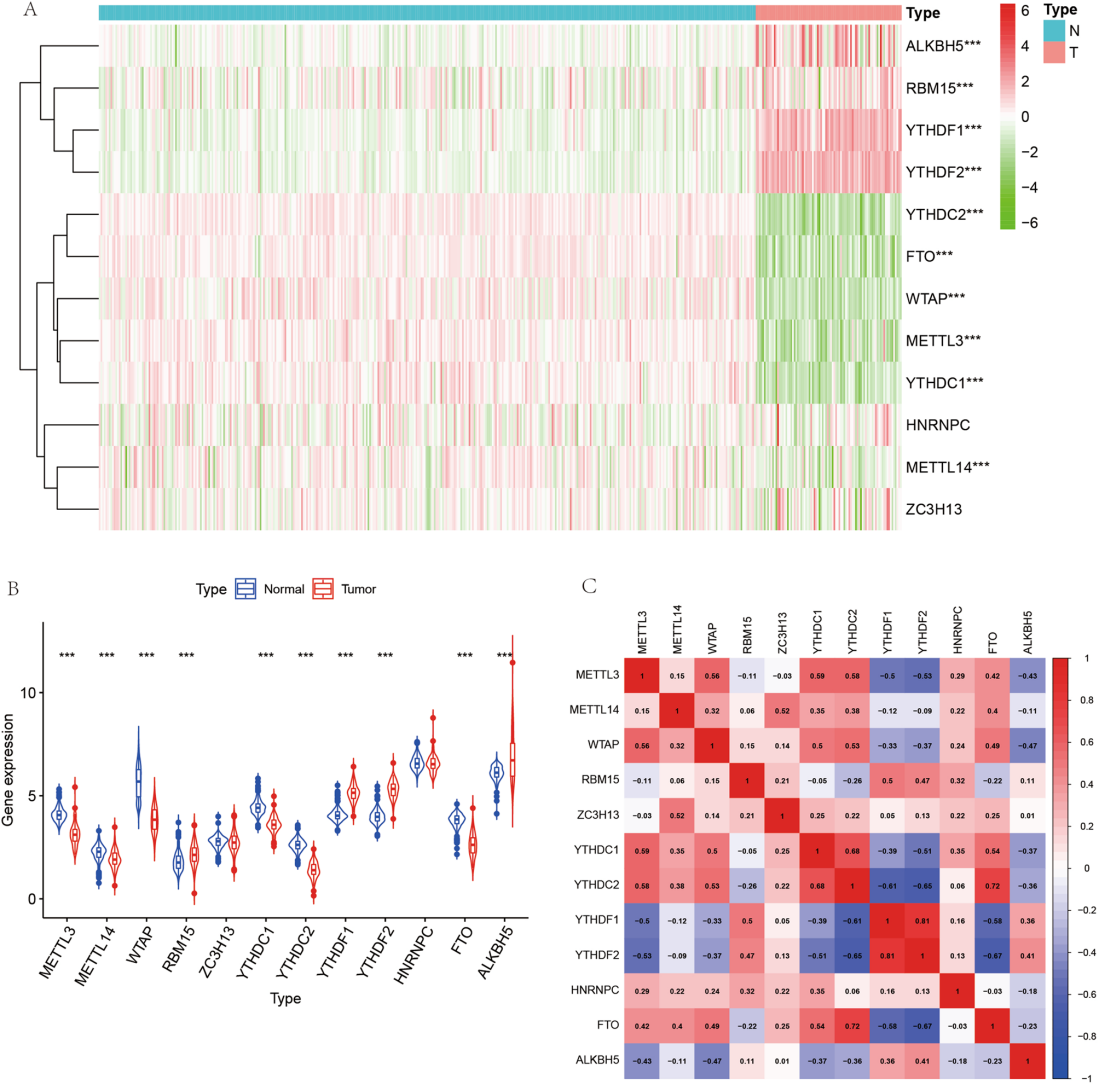
M6A-related genes differential analysis and correlation analysis plots. (**A**) Shows the heat map of m6A-related genes, red indicates high expression and green indicates low expression. "***" indicates P < 0.001. (**B**) Indicates the difference in expression of m6A-related genes in osteosarcoma and paraneoplastic tissues. (**C**) Indicates the correlation analysis graph between m6A-related genes.

We extracted m6A-related genes for further analysis to investigate the role of m6A-related genes in osteosarcoma. We extracted a total of 12 m6A-related genes from 54,751 rows of gene expression matrix for analysis. Subsequently, we performed differential expression analysis of these 12 m6A-related genes and plotted them into heat map (Fig. 2A) and violin map (Fig. 2B). The details of differentially expressed genes are illustrated in Table 2. Here, we constructed a correlation circle diagram between genes to analyze the correlation between these m6A genes (Fig. 2C). We made a two-by-two association of all m6A-related genes. If the line between two genes is red, the expression of these two genes in osteosarcoma is synergistically high, and if the line between two genes is green, the expression of the two genes in osteosarcoma is synergistically low.

**Table 2:** Details of the differentially expressed m6A-related genes.

Gene	conMean	treatMean	logFC	P value
YTHDC2	2.607617	1.396502	− 0.90092	3.73E−47
YTHDF2	3.996537	5.302091	0.407811	3.20E−46
WTAP	5.646791	3.828828	− 0.56053	3.86E−43
YTHDF1	4.081145	5.124077	0.328318	9.84E−43
FTO	3.807339	2.605391	− 0.54728	8.18E−42
YTHDC1	4.42701	3.589309	− 0.30263	6.82E−40
METTL3	4.086343	3.134333	− 0.38265	4.10E−37
METTL14	2.240601	1.919177	− 0.2234	1.19E−11
ALKBH5	6.109931	6.918332	0.179268	3.70E−09
RBM15	1.809804	2.148501	0.247497	4.28E−09
ZC3H13	2.760139	2.727128	− 0.01736	0.172505
HNRNPC	6.583267	6.571413	− 0.0026	0.520275

### Gene oncology enrichment analysis (GO) and protein–protein interaction network

**Figure 3:**
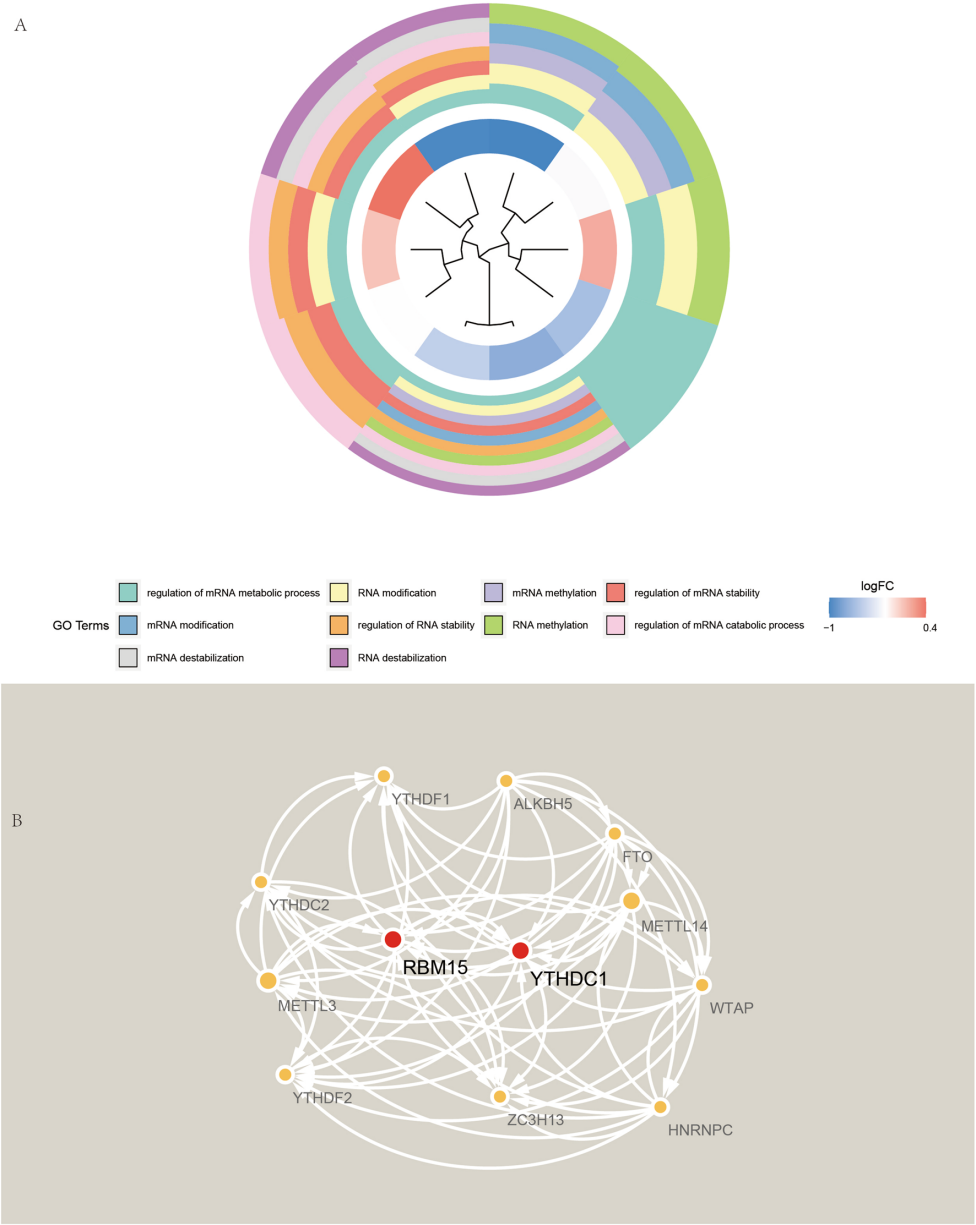
GO enrichment analysis and protein–protein interaction network diagram. (**A**) Shows the GO enrichment analysis of m6A-associated genes. (**B**) Shows the protein–protein interaction network of m6A-related genes.

Here, GO enrichment analysis was performed to analyze the function of GO of these m6A-related genes (Fig. 3A). The results of GO demonstrate that the first 10 items are: regulation of mRNA metabolic process, RNA modification, mRNA methylation, regulation of mRNA stability, mRNA modification, regulation of RNA stability, RNA methylation, regulation of mRNA catabolic process, mRNA destabilization, and RNA destabilization. In contrast, these m6A-related genes were imported into the STRING database, and their protein–protein interaction network diagram was obtained (Fig. 3B). In the figure we could find that RBM15 and YTHDC1 are each directly or indirectly linked to several m6A-related genes.

### m6A-related tumor typing and its correlation analysis

**Figure 4:**
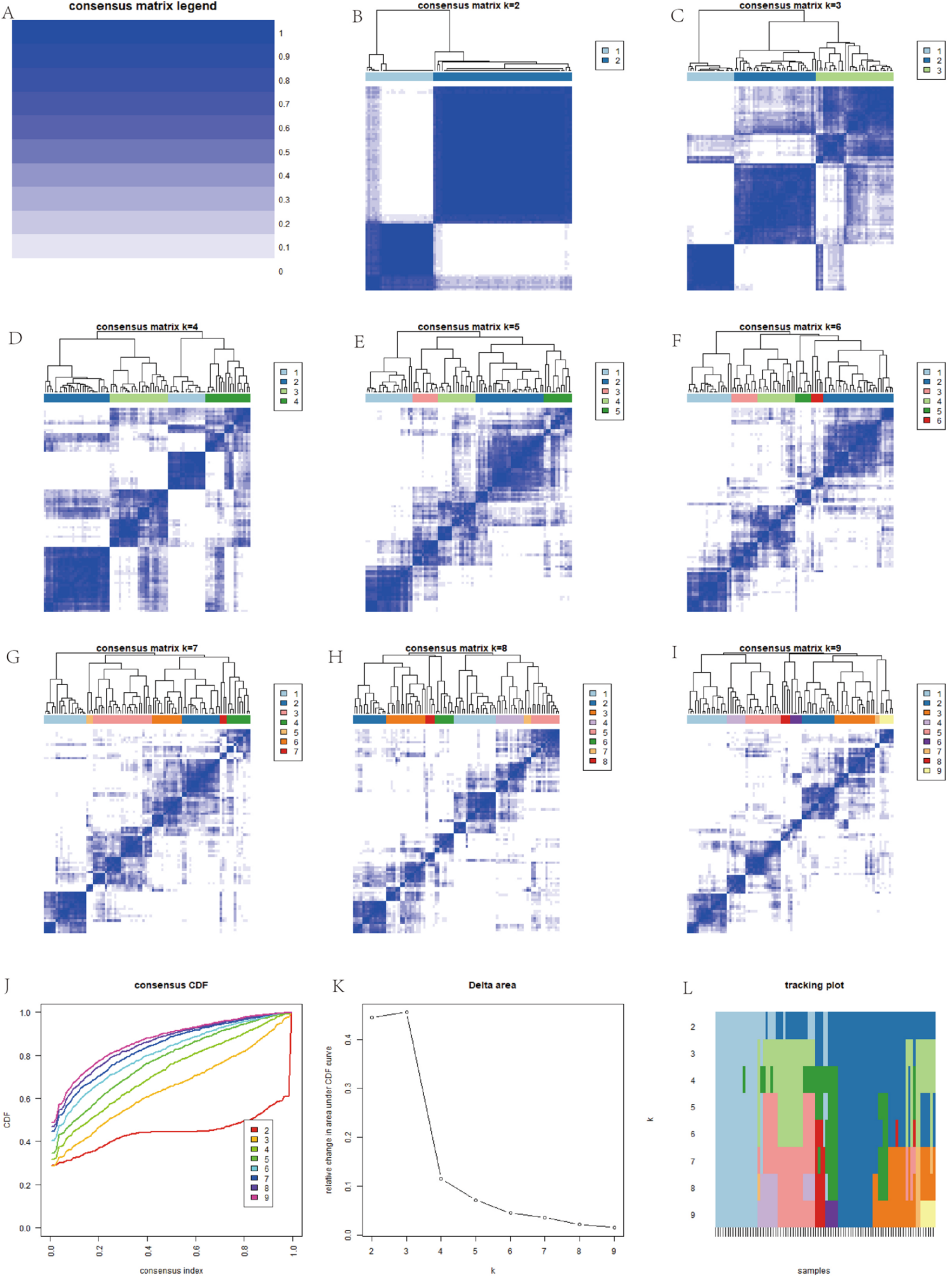
M6A-associated gene tumor typing map. (**A**–**L**) Indicates that we simulated the classification of tumors into 1–9 different grouping scenarios based on the expression of m6A-related genes.

**Figure 5:**
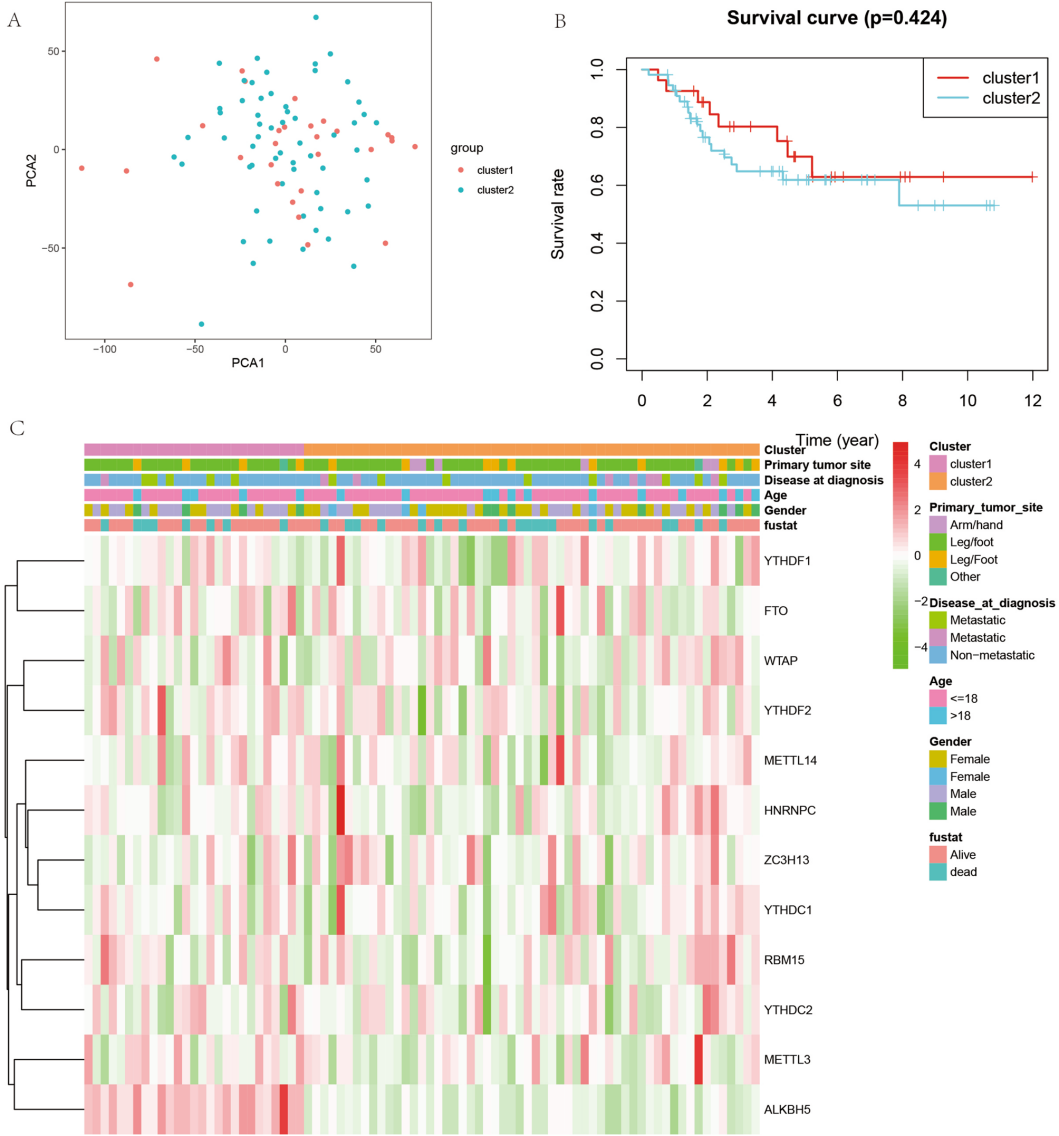
Principal component analysis, prognostic analysis and clinical correlation analysis of m6A-related genes based on tumor typing. (**A**) Shows the principal component analysis. (**B**) Shows the KM curves of the prognostic analysis constructed based on two different cluster groupings. (**C**) Represents the clinical correlation analysis based on two different clusters.

To analyze the role of m6A-related genes in osteosarcoma extensively, all cases were divided into different clusters of 1, 2, 3, 4, 5, 6, 7, 8, and 9 based on the simulation of these m6A gene expression values (Fig. 4A–L), and the CDF values were calculated. The best results were obtained when the CDF value was 2. Subsequently, a principal component analysis plot was constructed (Fig. 5A) by grouping all cases based on cluster1 and cluster2. Also, the difference in survival analysis based on m6A tumor staging (Fig. 5B) was not statistically significant (P > 0.05). We also analyzed the relationship of m6A-based tumor typing with clinical information (Fig. 5C).

### Survival analysis

**Figure 6:**
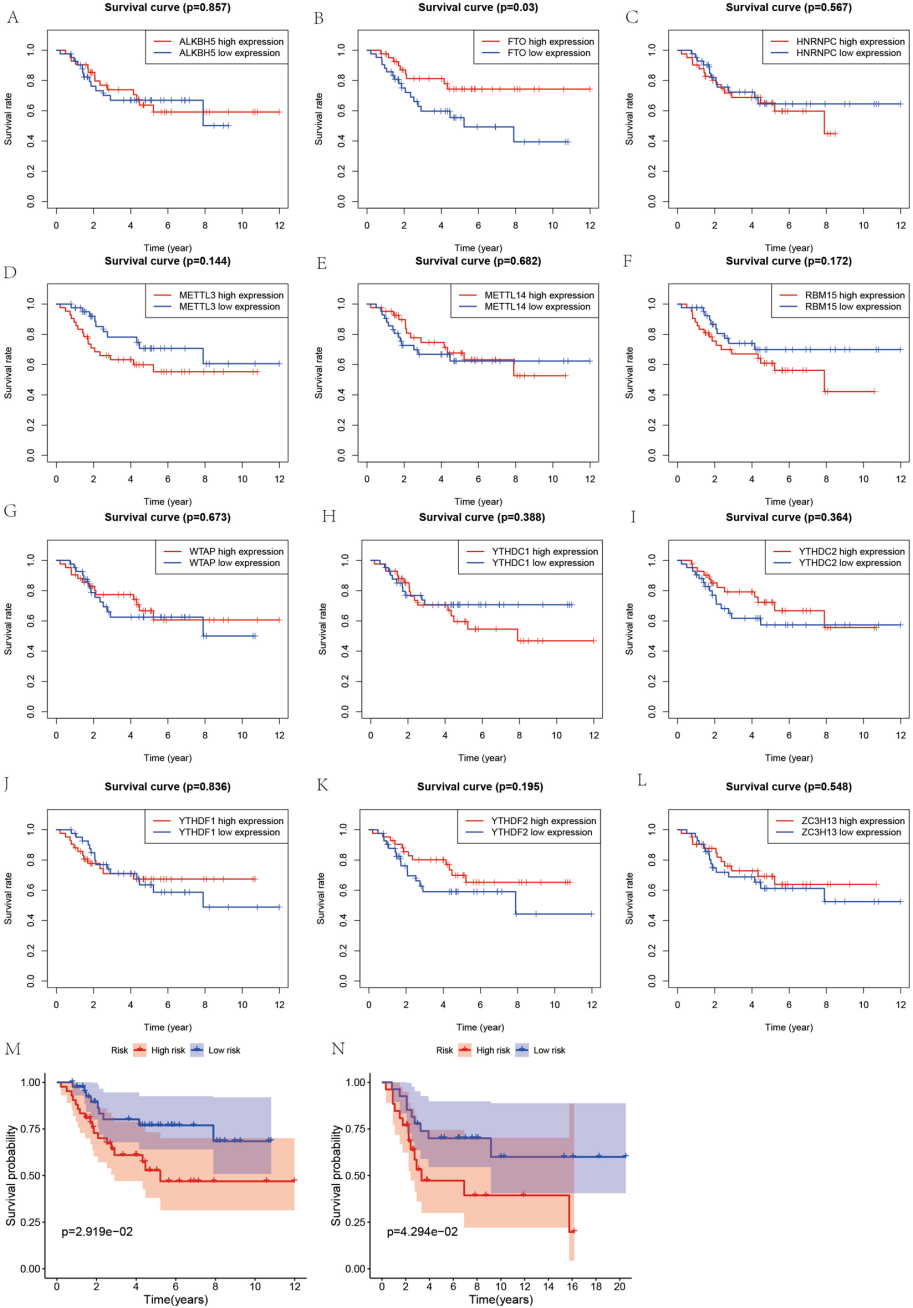
Survival analysis. (**A**–**L**) Shows the KM survival curves constructed based on the high and low expression of m6A-related genes in osteosarcoma and paraneoplastic tissues. (**M**) Shows the KM survival curves constructed based on the high and low risk groups of the m6A-related osteosarcoma prognostic model.

Here, two different methods were used to analyze the survival data of ghost sarcoma cases to obtain the best results. First, survival curves (Fig. 6A–L) were constructed based on the mean values of gene expression of these 12 m6A-related genes, dividing all patients into high and low expression groups. The results revealed that the differences were not statistically significant (P > 0.05). In contrast, the survival rate of patients in the high-risk group was much lower than that of those in the low-risk group by analyzing the differences between the high-risk and low-risk groups based on the constructed prognostic model of osteosarcoma with m6A-related genes (Fig. 6M), and the differences were all statistically significant (P < 0.05). Results from the validation dataset of the GEO database (Fig. 6N) showed that patients in the high- and low-risk groups constructed based on this prognostic model also had lower survival rates in the high-risk group than in the low-risk group in the GEO database (P < 0.05).

### Construction of m6A-related prognostic model for osteosarcoma

**Figure 7:**
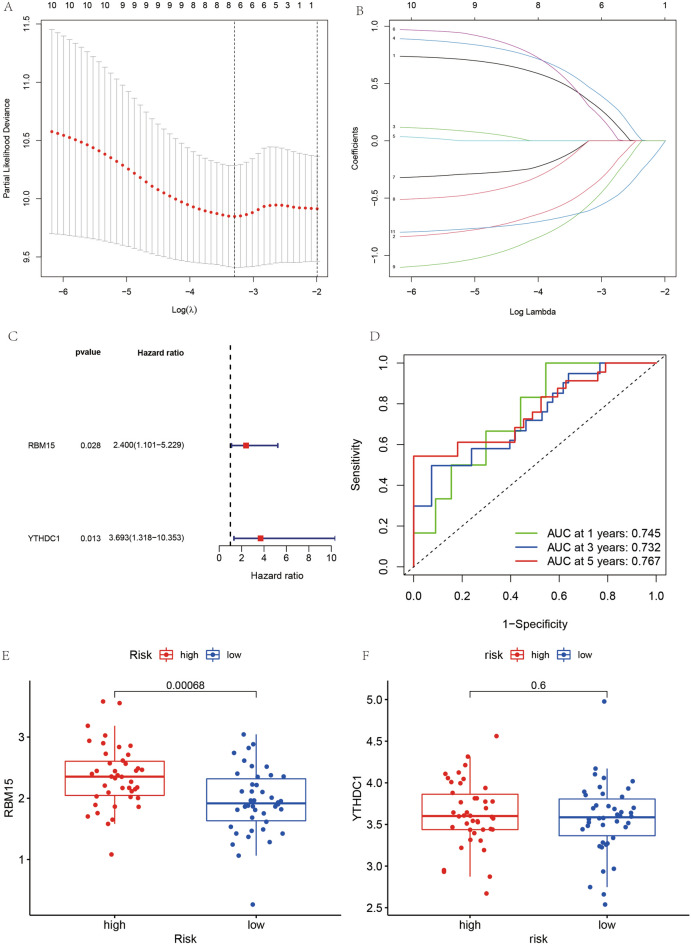
Construction diagram of the prognostic model for osteosarcoma. (**A**) and (**B**) represent the results of LASSO regression analysis to find the minimum penalty coefficient. (**C**) Shows the results of multivariate COX regression analysis, plotting the forest plot of 2 genes for constructing the prognostic model of osteosarcoma. (**D**) Represents the ROC diagnostic curve. (**E**) and (**F**) indicate the expression of the two genes used to construct the model in the high and low risk groups.

Since the results of the m6A-based model for tumor typing did not yield satisfactory results, a COX regression model was constructed. The m6A-associated genes were analyzed with survival by LASSO regression to obtain the minimum penalty coefficient (Fig. 7A,B). Subsequently, these genes were further analyzed concerning survival status and survival time using multivariate COX regression analysis, and finally, two genes (RBM15 and YTHDC1) were obtained for the COX prognostic model, which was visualized using forest plots (Fig. 7C). Moreover, the risk score of each patient was obtained; patients with greater than the mean value of risk score were categorized into a high-risk group and those with less than or equal to risk score into low-risk group.

### Examination of m6A-related prognostic models for osteosarcoma

Receiver operating characteristic (ROC) diagnostic curves were constructed (Fig. 7D) to be used to test the accuracy of the prognostic model. As illustrated by the ROC diagnostic curve, the area under the curve values are > 0.5 in predicting survival at 1 year or 3 and 5 years. This also demonstrates the accuracy of our constructed m6A-related prognostic model for osteosarcoma. In contrast, the expression of these two gene lists for the construction of the model in the high and low-risk groups was analyzed (Fig. 7E,F). The differences in gene expression of RBM15 in the high-risk and low-risk groups were determined, and the expression of RBM15 was significantly higher in the high-risk group than in the low-risk group, and the difference was statistically significant (P < 0.05).

### Prognostic prediction and clinical correlation analysis of m6A-related osteosarcoma prognostic model

**Figure 8:**
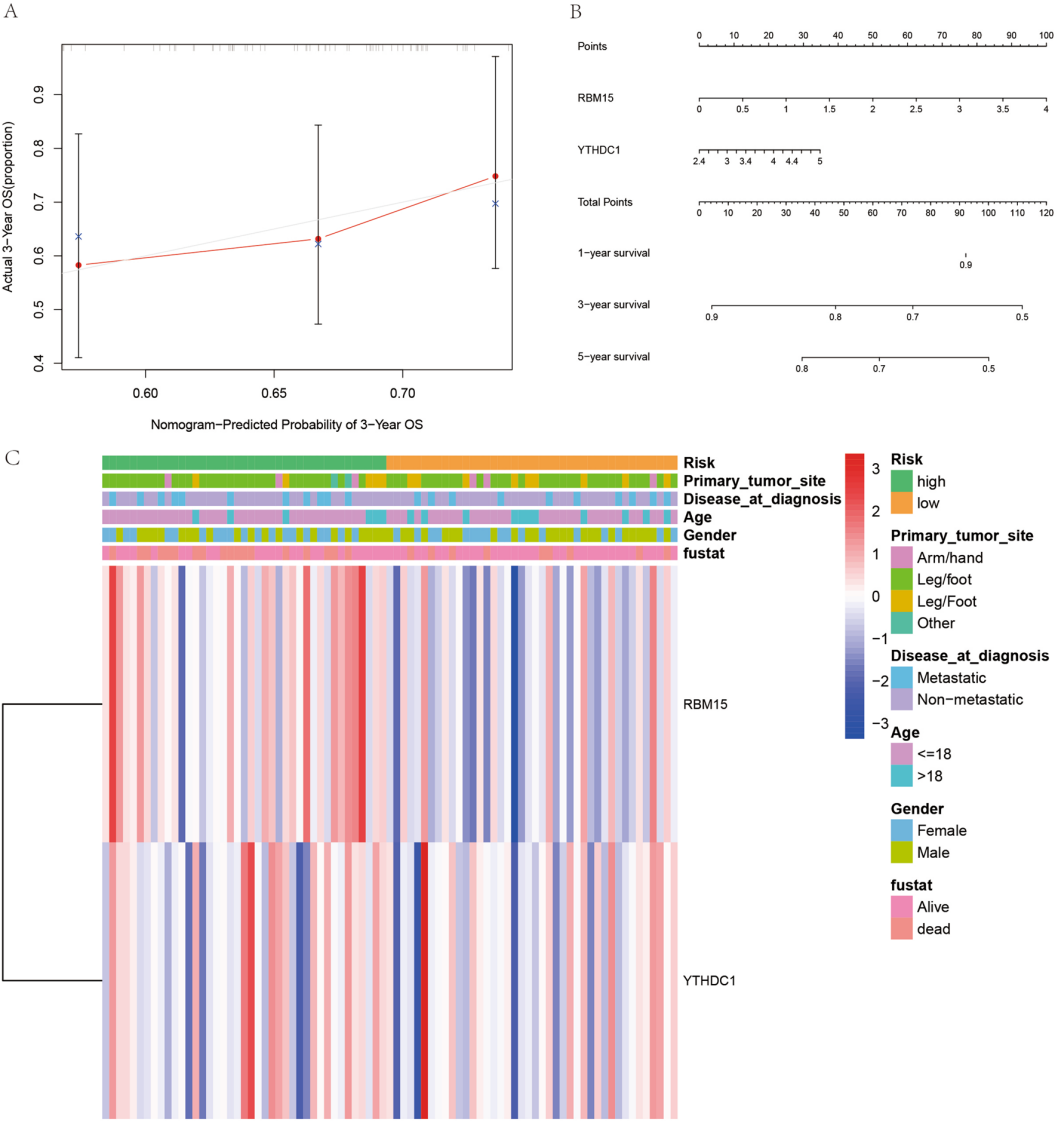
M6A-related osteosarcoma prognostic model validation and clinical correlation analysis. (**A**) Represents the calibration curve plot, the predicted starting point and the predicted end point basically overlap. (**B**) Represents the column line plot, which can be used to predict the prognosis of osteosarcoma patients. (**C**) Is the clinical correlation analysis plot.

A prognostic model was constructed for osteosarcoma for deriving the calibration curve (Fig. 8A) and nomogram plot (Fig. 8B) for predicting the prognosis of patients with osteosarcoma. The predicted value of the initiating point of the prediction overlaps with the actual value, and the predicted value of the focus of the prediction also basically overlaps with the actual value, which further confirms the accuracy of our constructed prognostic model. Also, the nomogram was used to predict the prognosis of patients with osteosarcoma. Finally, the relationship between risk values and clinical information was visualized by ranking patients in order from lowest to highest based on the risk score values (Fig. 8C).

### Immune cell composition analysis

**Figure 9:**
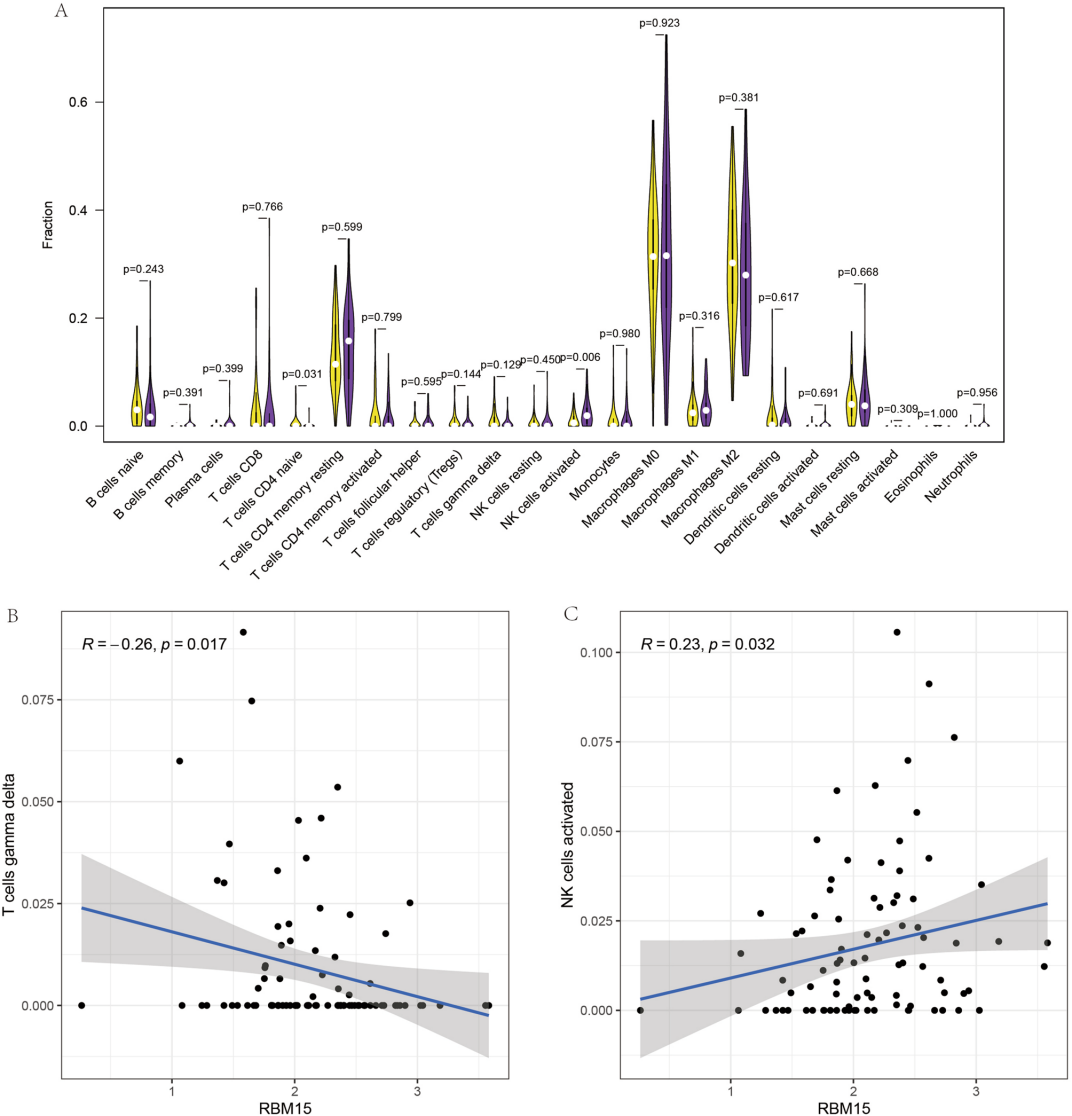
Immune cell composition analysis and correlation analysis of RBM15. (**A**) Represents the composition analysis graph of immune cells based on RBM15. (**B**,**C**) Show the correlation analysis between RBM15 and two different immune cells, respectively.

We quantified immune cells using CIBERSORTx software to analyze the relationship between two genes and immune cells in osteosarcoma using the constructed prognostic model (Fig. 9A–C). A significant negative correlation was detected between RBM15 and T cells gamma delta (R = − 0.26, P = 0.017). This would suggest that when the RBM15 gene is overexpressed, T cells gamma delta expression is reduced in osteosarcoma. On the other hand, a significant positive correlation between RBM15 and activated natural killer (NK) cells (R = 0.23, P = 0.032). This suggests a new direction for immunotherapy in the treatment of osteosarcoma. This suggests that when RBM15 gene expression is high, the expression of natural killer (NK) cells in osteosarcoma is also correspondingly increased.

### Drug sensitivity analysis

**Figure 10:**
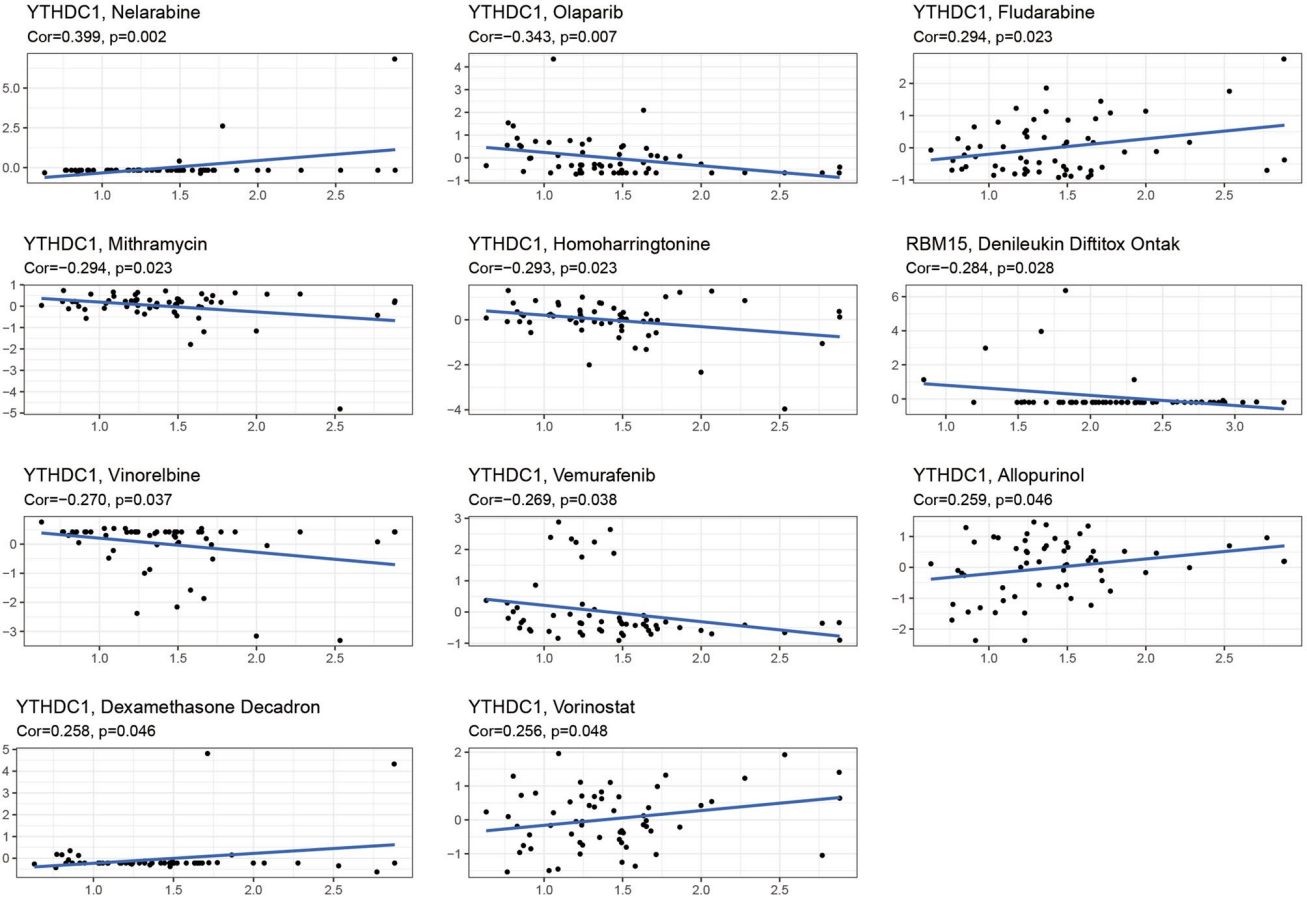
Drug sensitivity analysis. The graph shows the results of the analysis of gene expression and drug sensitivity, the horizontal coordinates indicate the gene expression values and the vertical coordinates indicate the Z-scroe values of sensitivity. Cor > 0 indicates a positive correlation and Cor < 0 indicates a negative correlation.

As shown in Fig. 10, we constructed the relationship between the gene expression of these two genes and the sensitivity of the drugs. From the figure, we could find that YTHDC1 showed a very significant correlation with Nelarabine, Olaparib, Fludarabine, Mithramycin, Homoharringtonine, Vinorelbine, Vemurafenib, Allopurinol, Dexamethasone Decadron and Allopurinol showed a highly significant correlation (P < 0.05). Among them, YTHDC1 showed positive correlation with Nelarabine, Fludarabine, Allopurinol and Allopurinol. In other words, if increased expression of YTHDC1 is detected in a patient's gene, then these drugs will be more sensitive than others for treating this patient. YTHDC1 showed a negative correlation with Olaparib, Mithramycin, Homoharringtonine, Vinorelbine and Vemurafenib, i.e. the higher the gene expression value, the weaker the drug sensitivity. On the other hand, RBM15 showed a negative correlation with Denileukin Diftitox Ontak (P < 0.05), i.e., the higher the RBM15 expression value, the worse the sensitivity of this drug. In other words, if an increased RBM15 is detected in a patient with osteosarcoma, then this patient is insensitive to Denileukin Diftitox Ontak.

### Immunohistochemical analysis

**Figure 11:**
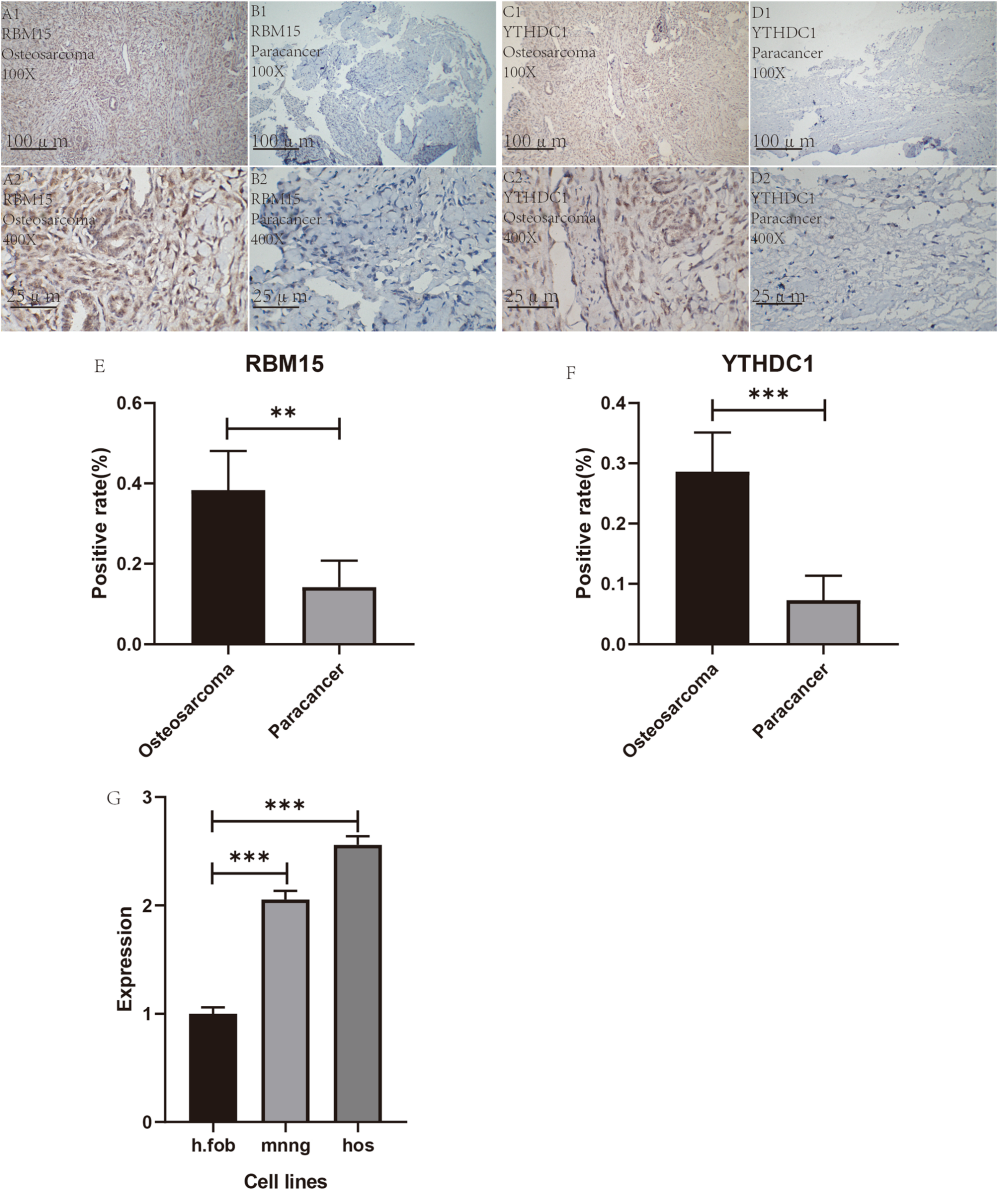
Immunohistochemical analysis and statistical analysis of positive rate. (**A1**–**B2**) Show the specific expression of RBM15 in osteosarcoma and in paraneoplastic tissues. (**C1**–**D2**) Show the difference of YTHDC1 expression in osteosarcoma and in paraneoplastic tissues. (**E**,**F**) Indicate the statistical plots of all positive immunohistochemical staining rates for RBM15, respectively. (**G**) Shows the expression of RBM15 in two osteosarcoma cell lines and one control cell line, "*" indicates P < 0.05, "**" indicates P < 0.01, "***" denotes P < 0.001.

The immunohistochemical analysis of osteosarcoma and paraneoplastic tissue specimens was performed at the First Clinical Affiliated Hospital of Guangxi Medical University that was used for pathological testing during the surgical treatment. Six pairs (six osteosarcomas and six paraneoplastic tissues) of pathological tissue sections were stained with specific antibodies for each gene, and a total of 24 sections were specifically stained. The results demonstrated that the positive area of specific staining expression of RBM15 was significantly more abundant in osteosarcoma than in paraneoplastic tissues (Fig. 11A1–B2). The positive area of specific staining expression of YTHDC1 was significantly more abundant in osteosarcoma than in paraneoplastic tissues (Fig. 11C1–D2). Also, we used Image J software to count the positive rate of the specifically stained sections for the RBM15 and YTHDC1 genes and then performed statistical analysis using a *t* test for the mean of two paired samples with IBM SPSS Statistics 25. Graphpad prism 8 (https://www.graphpad.com/guides/prism/8/user-guide/) was used to visualize the positive rate statistics for both genes. After performing statistical analysis, the rate of positive specific staining for RBM15 was higher in osteosarcoma than in paraneoplastic tissues (Fig. 11E), and the difference was statistically significant (P < 0.05). The positive rate of specific staining for YTHDC1 was higher in osteosarcoma than in paraneoplastic tissues (Fig. 11F), and the difference was statistically significant (P < 0.05).

### qRT-PCR

After cell culture, RNA extraction, primer design and QRT-PCR laboratory manipulation, we obtained the expression of RBM15 in osteosarcoma cells as well as in control cells. From Fig. 11G, we can find that the expression of RBM15 in both osteosarcoma cell lines was significantly higher than that in the control group, and the difference was statistically significant (P < 0.001). This further tested the accuracy of our analysis at the cellular level.

### Big data blood routine data results

**Figure 12:**
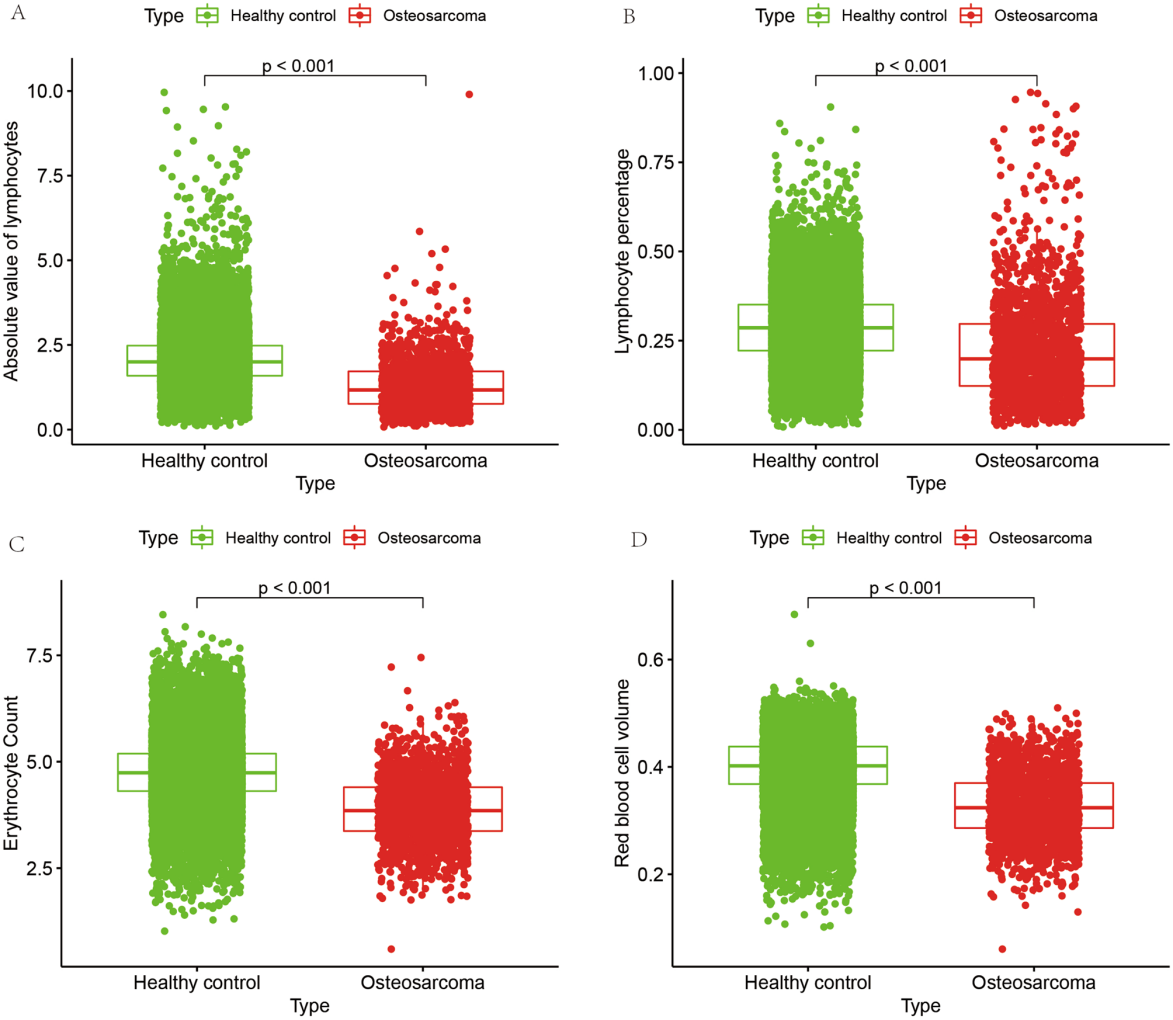
Results of routine blood analysis of big data. (**A**,**B**) Demonstrate the differences in absolute lymphocyte values and lymphocyte percentages in the osteosarcoma and control groups, respectively. (**C**,**D**) Show the differences in red blood cell count and red blood cell volume between the osteosarcoma and control groups.

To test the accuracy of immune cell differences obtained from bioinformatics CIBERSORTx analysis, we collected data on routine blood tests from 1738 patients diagnosed with osteosarcoma and 24,344 non-osteosarcoma patients (healthy controls) at the First Affiliated Hospital of Guangxi Medical University from January 1, 2010 to January 1, 2022, for a total of 26,082 cases in all cases. We collected four indices of routine blood data: absolute lymphocyte value, lymphocyte percentage, red blood cell count and erythrocyte specific volume. The results (Fig. 12A–D) showed that the absolute lymphocyte value, lymphocyte percentage, hematocrit and erythrocyte count were lower in osteosarcoma than in the control group (P < 0.001). This further validates the reliability of our analysis. The previous bioinformatics analysis also revealed significant differences between these immune cells in the osteosarcoma and control groups.

## Discussion

Osteosarcoma as a soft tissue sarcoma has a poor prognosis, especially the highly aggressive soft tissue sarcomas^21^. The protein function that specifically recognizes m6A reveals that it is a modification used by cells to accelerate mRNA translation and metabolism^22^. Since 1974, m6A RNA methylation has been identified as a major internal modification of higher eukaryotic RNAs. For different tissues and cell lines, m6A methylation is capable of influencing tumorigenesis and development through various mechanisms^23^. Moreover, in the present study, the results of GO enrichment analysis showed that these m6A-related genes were primarily enriched in regulation of mRNA metabolic process, RNA modification, mRNA methylation, regulation of mRNA stability, mRNA modification, regulation of RNA stability, RNA methylation, regulation of mRNA catabolic process, mRNA destabilization, and RNA destabilization entries. This is consistent with the results of our analysis. Also, the results of both protein–protein interaction network and intergenic correlation analysis demonstrated that these m6A-related genes were closely linked.

RBM15, a protein-coding gene, is associated with the RBM15 gene in diseases such as acute megakaryocytic leukemia and megakaryoblastic acute myeloid leukemia With T(1;22)(P13; Q13). RBM15, a type of m6A methyltransferase, has growing evidence that m6A methylation has a dramatic impact on RNA and is involved in the pathogenesis of a variety of diseases, including cancer^24^. Also, dysregulation by m6A modification and its associated proteins contributes to cancer development, occurrence, and drug response^25^. Furthermore, m6A modifications play a unique role in critical physiological functions of the liver and various liver diseases. Dynamic post-transcriptional modifications determine the fate of target RNAs by regulating various aspects of RNA processing, including RNA export, transcript processing, splicing, and degradation, while the most abundant internal mRNA modifications in eukaryotic cells are by using m6A, which plays an important physiological role in carcinogenesis and embryonic development^26^. Moreover, in the present study, we showed through bioinformatics with laboratory level validation results that RBM15 plays an integral role in osteosarcoma and that a prognostic model of osteosarcoma consisting of two genes, including RBM15, was able to adequately predict the prognosis of patients with osteosarcoma. It has been shown that in a mouse model of spontaneous breast cancer, tumors can maximize the chance of metastasis through a systemic validation cascade of gas exchange and that tumor-induced neutrophils can acquire the ability to carry cytotoxic T lymphocytes transporting CD8 antigens^27^. Gamma delta (γδ) T cells are recognized as protective cells in cancer, primarily through the production of potent cytotoxicity and interferon-γ^28^. There is growing evidence for a role of γδ T cells as additional drivers of tumor development, and these native γδ T cells are abundant in both mouse and human tumor microenvironments^29^. This is consistent with our findings. Our study showed that γδ T cells in osteosarcoma are closely associated with RBM15. NK cells can rapidly kill multiple adjacent cells as long as they exhibit surface markers associated with oncogenic transformation, a unique property of immune cells that enhances the ability of antibodies and T-cell responses to support the role of NK cells as anti-cancer agents^30^. Chimeric antigen receptors (CARs) can significantly increase the antitumor activity of immune effector cells and improve NK cell-mediated killing^31^. This is consistent with the results of this study, which showed a significant positive correlation between RBM15 and NK cell activation in osteosarcoma. Finally, we found that the expression of RBM15 in both osteosarcoma cell lines was much higher than that in the control cell lines by qRT-PCR in vitro, and the difference was statistically significant (P < 0.05).

YTH Domain Containing 1 (YTHDC1) is a protein-coding gene, and diseases associated with the YTHDC1 gene include Wilms tumor 1 and periosteal chondrosarcoma. Wen Xiao et al. showed that due to YTHDC1 depletion, its dysregulation can be restored by wild-type but not m6A binding-deficient YTHDC1 recombination, demonstrating that the m6A reader YTHDC1 can exert regulatory mRNA splicing by recruiting and regulating pre-mRNA splicing factors and permitting splicing factors to enter the binding region of target mRNAs splicing role^32^. Also, double-strand breaks (DSBs) have been demonstrated as the type of DNA damage that is optimally returned and may lead to cell death or genomic instability if not repaired and that the METTL3-m6A-YTHDC1 axis can regulate the accumulation of DNA-RNA hybrids at DSBs sites. Moreover, depletion of METTL3 markedly enhanced the sensitivity of mouse xenografts and cancer cells to DNA damage treatment^33^. This is consistent with the results of the present study. In this study, bioinformatics with laboratory level studies showed that the prognostic model of osteosarcoma consisting of two genes, including YTHDC1, demonstrated a close correlation with the prognosis of patients with osteosarcoma whose survival rate was much lower in the high-risk group than in the low-risk group.

Here, prognostic models were constructed by two different methods. First, tumors were typed based on the expression of m6A-related genes, and all osteosarcoma cases were divided into two clusters. Based on this, PCA plots, survival analysis, and clinical correlation plots were constructed. Subsequently, LASSO regression and multivariate COX regression analysis were used to construct a prognostic model for m6A-related genes to construct a more accurate model. The results showed that the survival rate of patients with osteosarcoma in the high-risk group was much lower than that in the low-risk group. Results from the validation dataset of the GEO database showed that patients in the high- and low-risk groups constructed based on this prognostic model also had lower survival rates in the high-risk group than in the low-risk group in the GEO database (P < 0.05). The ROC diagnostic curve and calibration curve was tested on the model, and the prognosis of osteosarcoma cases could be predicted using nomogram plots. Subsequently, an analysis of the immune cell composition of these two genes was performed, which showed that RBM15 was closely associated with both immune cells, suggesting a new direction and perspective for immunotherapy of osteosarcoma. Finally, the difference in protein expression of these two genes was analyzed in osteosarcoma tissue and normal para cancerous tissue by immunohistochemistry, and the results supported this analysis. Subsequently, we examined the RBM15 gene by qRT-PCR using in vitro cellular assays and found that its expression was indeed higher in the osteosarcoma cell line than in the control cell line, which further confirmed the accuracy of our analysis. More notably, we collected 1738 patients diagnosed with osteosarcoma and 24,344 non-osteosarcoma patients from January 1, 2012 to January 1, 2022 at the First Affiliated Hospital of Guangxi Medical University to verify the accuracy of CIBERSORTx analysis of immune cell differences using two independent sample *t* tests, and the results also confirmed that our bioinformatics analysis was very accurate.

Our study also has certain shortcomings. First, the sample size was inadequate. Our analysis was based on two databases of 88 osteosarcoma samples and 396 normal samples, which was inadequate. Second, laboratory level validation was inadequate. We only performed preliminary validation of the results of the analysis by immunohistochemistry and did not perform in-depth laboratory validation.

## Conclusion

RBM15 could be possible m6A-related biomarker for predicting the prognosis of osteosarcoma with much lower survival rates in the high-risk group than in the low-risk group.

## Data Availability

The datasets supporting the conclusions of this article are available in the UCSC Xena (http://xena.ucsc.edu/), GTEx database (https://www.gtexportal.org/home/), and GEO database (https://www.ncbi.nlm.nih.gov/geo/query/acc.cgi?acc=GSE21257).
